# Gender Differences in the Experience of Infertility Concerning Polish Couples: Preliminary Research

**DOI:** 10.3390/ijerph16132337

**Published:** 2019-07-02

**Authors:** Małgorzata Nagórska, Anna Bartosiewicz, Bogdan Obrzut, Dorota Darmochwał-Kolarz

**Affiliations:** 1 Institute of Experimental and Clinical Medicine, Medical Faculty, University of Rzeszow, 35-959 Rzeszow, Poland; 2 Institute of Nursing and Health Sciences, Medical Faculty, University of Rzeszow, 35-959 Rzeszow, Poland; 3 Department of Obstetrics and Gynecology, Clinical Provincial Hospital No.2, Rzeszow, 35-301 Rzeszow, Poland

**Keywords:** infertility, treatment, psychosocial problems

## Abstract

The World Health Organization (WHO) determines infertility as a disease of the reproductive system defined clinically by the failure to achieve a clinical pregnancy after 12 months or more of regular unprotected sexual intercourse. Estimates indicate that the problem of infertility in the world is continuing to grow. The aim of the study was to compare approaches to disease in partners of both sexes diagnosed with infertility. The study was conducted among 61 couples treated for infertility using an original questionnaire developed by the authors. The Chi square independence test was used for statistical analysis. Both men and women responded to the diagnosis of infertility with negative emotions. Regardless of sex, sadness and anxiety were the dominant feelings associated with the diagnosis of infertility. Women believed in the success of the treatment to a greater extent than men. Mainly women attempted to talk openly about the problem of infertility, while men were more restrained in this respect. Women accepted the assisted reproductive technologies (ART) to a greater extent than men, but men would accept childlessness more often than women.

## 1. Introduction

The World Health Organization (WHO) provides a clinical definition of infertility as “a disease of the reproductive system defined by the failure to achieve a clinical pregnancy after 12 months or more of regular unprotected sexual intercourse” [1].

According to the available estimates, the problem of infertility is growing in the world. Lewine et al. state that their extensive meta-regression analysis outlines a remarkable decrease in sperm counts between 1973 and 2011, with a 50–60% decline in the group of fertile men from Europe, North America, Australia, and New Zealand [2]. Precise estimation of the population of infertile patients is difficult for three reasons: the difference in definitions of infertility (1, 2, or 5 years of attempts to conceive), significant difference in selected populations (large populations vs. epidemiological studies) and specifying whom the diagnosis involves (women, couples, or individuals) [3].

In 2010, the number of infertile couples was estimated at 48.5 million (about 24 million infertile women), i.e., an increase of 6 million couples since 1990 [4]. According to Rustein and Shah, 186 million married women from developed countries (excluding China) were infertile in 2002 [5]. This discrepancy results from different time and place of estimates and the use of different calculation methods, as well as from differences in the age of the assessed population. Mascarenhas et al. limited their study to women aged 20–44 years, while Rustein and Shah studied women aged 15–49 years. According to the data of the Polish Gynecological Society, 1.5 million couples have problems with conceiving offspring in Poland [6].

Current data on the prevalence of infertility among women of reproductive age indicates that it occurs in every seventh couple in Western countries, while every fourth couple in developing ones is affected. In some regions of the world (Southeast Asia, North Africa, and Central and Eastern Europe), it may be as high as 30% [4].

Infertility should be considered not only as a medical or psychological problem, but also a social one due to the inability to fulfill one of the basic social roles—parenthood. It is explained as follows: the result of the biomedical problem is disease, disability, and the feeling of being unwell in the case of an infertile couple; however, both partners may feel perfectly well, but in trying to conceive offspring with their partner, they create a new quality of “a patient”—the infertile couple. It is quite likely they would never have such a problem in relation with another person [7].

Infertility treatment imposes a physical, mental, and emotional burden. Infertile couples struggle with stress; the literature describes this as a “crisis of infertility” [8]. This crisis is quite complex, because it involves reactions regarding the medical procedures necessary for diagnosis and treatment, reactions of others, readiness to be parents or not, and individual characteristics of the partners. Problems stated concern both the couple and each partner individually.

Procedures of diagnostics and treatment of infertility are very expensive. Many infertile couples spend a significant part of their income on treatment, which imposes a heavy burden on the home budget. In health economics, such a situation is referred to as “catastrophic expenses”. By definition, these include every expense for treatment from out of pocket that threatens the survival of the household by exceeding 40% of annual food expenditure [9].

In many cases, infertility treatment is a long-term and expensive therapy requiring numerous sacrifices and subordinating life to medical procedures. Such a situation also imposes a burden on the psychosocial sphere. Men and women experience this problem differently. Many authors emphasize that experiencing infertility is one of the most stressful events in a couple’s life [10,11,12,13,14,15,16].

In our paper, we wanted to draw attention to the complexity of nonmedical problems accompanying infertility treatment and the differences between women and men in the approach to treatment and coping with the disease.

### Aim

The aim of study was to compare approaches to disease of partners of both sexes diagnosed with infertility.

## 2. Materials and Methods

The research was conducted among infertile couples in one of the institutions dealing with infertility treatment in southeastern Poland. The inclusion criteria were infertility diagnosed in the couple according to the WHO clinical definition and voluntary participation in the study. The presented study is a part of a bigger project which we divided into two stages: The first stage was preliminary research, in which we tested the self-administrated questionnaire. The result of the first stage is described in this paper. In the second stage, we will analyze the quality of life and the level of satisfaction with life in infertile couples using standardized tools (Ferti-QoL and SWLS).

In our study, we used the authors’ questionnaire with closed-ended questions. Respondents chose from already-prepared answers (i.e., single-answer multiple-choice question) or had the option to add their own short answer (the “other” answer option at the end). The questionnaire consisted of three parts. The first part was addressed to both partners and contained 9 questions about the situation in the relationship and the course of previous treatment. The second part, intended for women, contained 17 questions about subjective feelings accompanying treatment and 5 questions about sociodemographic data. The third part, addressed to men, contained similar questions.

### 2.1. Ethical Consideration

The study was conducted in accordance with the Declaration of Helsinki for medical research. The authors obtained permission of the Bioethical Committee research at the University of Rzeszow (approval number 2018/04/12). All participants were notified about the possibility of withdrawing from the study at every stage.

### 2.2. Course of the Study

Surveys were handed to couples immediately after the appointment at a gynecologist. At the beginning, the participants were informed about the purpose of the study, anonymity and voluntary participation in the study. The partners jointly filled in the common part of the survey (part A), and then the interviewer asked for the separate completion of the other parts by the woman (part B) and the man (part C). After completing the questionnaire, all three parts were stapled together. The study included 80 couples diagnosed with infertility, of which 61 fully completed questionnaires were included in the final analysis (76%). With such a trial size, the test power was 0.87, which should be considered satisfactory (0.80 is assumed to be the lowest), taking into account the preliminary nature of the research. The result rightly suggests an increase in the sample size, which is planned in the extended research. Statistical analysis was performed using the STATISTICA 8.0 software (Statsoft Polska, Kraków, Poland). The results obtained were subjected to statistical analysis using the Pearson Chi square test. The level of significance was adopted at *p* < 0.05.

## 3. Results

In total, 61 couples (*n* = 122) treated for infertility participated in the study. The largest group among the respondents were people between 31 and 40 years of age. Overall, 77.2% of women and 52.5% of men surveyed had higher education. The majority of the respondents were urban residents (67.2%), and one-third lived in a rural area. Everyone declared that they were Roman Catholics. Two couples (3.2%) had children, but in both cases, they were the women’s children from previous relationships (Table 1).

All respondents were in a permanent relationship, of up to 5 years in the case of 44.3% of the respondents, from 6–10 years for 45.9%, and over 10 years for 9.8% of the couples. The couples who had attempted to conceive for 1–5 years accounted for 80% of the respondents, and every fifth couple (19.7%) had attempted to conceive for more than 5 years.

A significant proportion (62.3%) of the respondents knew the cause of their infertility; however, every third couple had not yet been diagnosed (37.7%). Among the reasons given, the largest proportion of male causes were abnormalities in the composition of the sperm, while in females, they were endometriosis, Polycystic Ovary Syndrome (PCOS), and fallopian tube obstruction.

Regarding previous treatment, every fifth couple (21.3%) had never used assisted reproduction techniques (ART). In the case of the remaining couples, insemination had been most often used, by 65.5%, followed by in vitro with micromanipulation (intracytoplasmic sperm injection, ICSI) by 8.2% and classic in vitro by 4.9%. The majority of the surveyed women (96.8%) had never been pregnant, while 11.5% had been pregnant but had miscarried. All men surveyed were childless.

According to 77% of the respondents, the costs of treatment were too high and over half of them (52.4%) spend all their savings on this treatment. In order to obtain funds for treatment, some of the respondents had taken an additional job or a loan from a bank or borrowed money from relatives. However, financial resources were not the only difficulty encountered after the decision to start the treatment (Table 2).

In most cases, there was no prior history of infertility in the respondents’ families. Problems with procreation in the immediate family occurred only in 8.2% of women and 6.6% of men. Statistical significance was found between the spouses’ knowledge of infertility in the family and their sex. Women had greater knowledge in this area (*p* = 0.050).

The response to the diagnosis of infertility, both in women and in men, was negative and was mostly sadness (32.8% of women and 34.4% of men) and helplessness (16.4% of women and 18.0% of men). Anxiety and fear appeared quite often in both sexes (22.9% of women, 14.8% of men). There was no statistical dependence found in this regard (*p* = 0.228).

According to 49% of the surveyed women and 45.9% of men, problems with procreation did not change their relationship. Every third couple (29%) declared that the problem and the need to seek help positively influenced the relationship and even brought the partners closer. There was no gender dependence in the relationship assessment (*p* = 0.328).

The greatest difficulty in treatment for women was the subordination of life to one goal—the treatment (42.6%)—which was reported as such by half as many men (19.7%) (*p* = 0.021). Longer procedures (34.4%) and embarrassing tests (21.3%) were more troublesome for men, which were much less frequently mentioned by women (16.4% and 8.2%, respectively). Long attempts to conceive resulted in a change of the existing lifestyle in 55.7% of women and 44.3% of men. These changes mainly concerned changes in diet and restriction of stimulants and ways of spending free time, as well as limiting social contacts.

Talking about the infertility problem and treatment significantly differentiated between the examined women and men (*p* = 0.001). Women were more willing to openly discuss the problem. These were primarily conversations with the spouse (42.6%) and friends (26.2%). In contrast, men primarily wanted to talk to their partners (68.8%), and medical staff were mentioned much less often (16.4%). They completely omitted friends and colleagues. Difficulties occurring during treatment were discussed only sporadically with parents or siblings, although half of the respondents (59% of women and 49.2% of men) declared their families’ acceptance of them taking up the treatment.

Women’s families more often accepted the treatment (49%), but every third family did not accept all methods (23%). In the case of men, every second family accepted the treatment, but there was no acceptance for some ART methods in up to 60% of them.

Over 70% of the couples fully accepted all ART methods; nearly 30% allowed only some of them as a method of treatment (*p* = 0.979). Most often, they did not agree with in vitro techniques with micromanipulation—intracytoplasmic sperm injection (ICSI)—accounting for 16.4% women and 11.4% men, respectively. Classic in vitro—in vitro fertilization (IVF)—was also not accepted by 14.7% women and 4.9% men.

Information about pregnancy or childbirth in relatives and friends often reminded the couples about their own problems and evoked emotions. Sadness was the most common feeling accompanying this situation—felt by 41% of women and 21% of men—and every third person felt joy.

The possibility of not having biological offspring was taken into account by 24.6% of women, while men were a much larger group (41%). Finally, regarding looking for other solutions to unintentional childlessness, 36.1% of women and 26.2% of men declared the possibility of adopting a child. Considering adoption, respondents of both sexes were most afraid of unpredictable problems with adoptive offspring in the future, while a less often declared obstacle was the long-term adoption procedure and the lack of acceptance of the immediate environment.

Most women and men (57% of women and 60% of men) expressed the opinion that couples affected by infertility should be covered by professional psychological assistance, but only in justified cases.

Analysis of the individuals’ approach to treatment and belief in its success demonstrated statistically significant differences in terms of sex (*p* = 0.017). Overall, 75.4% of women and 83.6% of men believed in the success of the treatment. Almost every third woman (24.6%) takes the treatment, although not believing in its success. A small proportion of the men (6.6%) were not convinced of its chances of success, but nevertheless made the effort for their wife (Table 3).

## 4. Discussion

Infertility treatment is usually a long and complicated process that does not always end successfully. An additional difficulty is the fact that infertility is a problem for two people and that the disease may have a multifactorial background, which is not always easy to determine [17].

In the examined group of women, the most common causes of infertility were endometriosis, polycystic ovary syndrome (PCOS), and fallopian tube obstruction, which was in line with the results of Bakunowicz et al. [18]. Surveyed males as the cause of failure most often indicated incorrect sperm parameters, which did not differ from the results of other authors; e.g., in a multicenter study from Poland, diagnosed causes of infertility in women were, in descending order of frequency, ovulation disorders, uterine factor, PCOS, fallopian tube factor, and endometriosis, and in males were sperm abnormalities, most often asthenozoospermia and asthenoteratozoospermia [17].

As the results of previous studies by other authors indicate, women and men have different approaches to the diagnosis, prognosis, and treatment of infertility. Women, for example, are more willing to talk about the problem. As our research has shown, women were more likely to benefit from social support networks than men; 42.6% of them frequently talk with their husband/partner, and they also significantly more often talk with friends (26.2%). In the Turkish study, Karaca obtained similar results, observing that the most important source of support for women (96%) is their husband [19]. Respondent men share their problem outside the home to a much lesser extent (1.6%); the greatest source of support for them is their wife/partner (68.8%), with a doctor in second place (16.4%). A Danish study confirmed this observation, finding that women often talk about infertility outside the home, while men prefer to discuss the problem with their wife and close family [20]. In turn, Martins et al. indicated that when infertile women are supported by their husbands, this improves communication in a relationship and also has a positive effect on the sex life of the couple [21].

In Wdowiak’s research, 29% of the infertile Polish couples claimed that the relationship did not deteriorate from the moment of infertility being diagnosed, while in the Makara-Studzińska study, this was the case for 37% of the respondents [22,23]. Similarly to our analysis, 49% of women and 45.9% of men indicated that their relationships have not changed. However, in two other studies, a positive influence of the couple’s infertility on the relationship was recorded [20,24]. In contrast, in Filipek’s study, the majority of respondent couples (58.3%) indicated mutual emotional distance and a lack of satisfaction with intercourse (60.8%) [25].

The diagnosis and treatment of infertility are accompanied by negative emotions; women, like men, most often feel sadness, grief, fear, and anxiety, and in addition, men pointed to disappointment. This is confirmed by the study by Bakunowicz et al., where anxiety, depression, frustration, and anxiety prevailed in the group of infertile women, while surprise, sadness, and anger dominated among infertile couples in Jędrzejczak’s study [18,26].

As it turns out, having your own child is more important for women than for men. In our analysis, every fourth woman (24.5%) and every second man (49.0%) was ready to accept the lack of an offspring. Similarly, in Pash’s study, having a child was found to be more important to wives than husbands [24].

In a situation when couples cannot have their own offspring, 36% of women and 26% of men surveyed would decide to adopt. In Saudi Arabian research, adoption would be accepted by 60.6% of infertile patients undergoing IVF and 71.5% of fertile patients [27].

The diagnosis of infertility is associated with a lot of stress, and therefore more attention needs to be given to the mental health needs of these patients and their partners [28]. In the case of infertile women, the level of stress is much higher than in men [10]. In the studies of Wdowiak and Makara-Studzińska, Polish respondents pointed to the need to use psychological assistance [22,23]. In addition, they believe that psychological support should be offered at the stage of diagnosing infertility [23]. According to our research, 42.6% of women and 34.4% of men claim that psychological assistance should be provided to all infertile couples. Psychological support for infertile couples is recommended by the American Society for Reproductive Medicine (ASRM) and the European Society for Human Reproduction and Embryology (ESHRE) [29].

The results of our research showed that men and women treat the diagnosis and treatment of infertility in different ways. Women were more committed, but at the same time, they were more emotionally concerned with this problem.

The treatment is an additional financial burden [30]. The research by Wdowiak et al. shows that the surveyed most frequently experienced emotional (53.64%) and financial (49.09%) problems during infertility treatment [22]. The economic problems were also indicated by 53% of infertile women in the study by Jędrzejczak, which is in line with our research, where 77% of the couples indicated that financing the treatment was a problem for them [26].

Our study confirmed the existence of gender differences in the approach to diagnosis and treatment of and coping with infertility. The results obtained may be useful for professionals dealing with the treatment of infertile couples and may contribute to the improvement of the quality of care and support provided to people struggling with the problem of unintended childlessness.

## 5. Conclusions

The biggest difficulties for men during the infertility treatment were the long process of the treatment and embarrassing tests, whereas for women, it was the subordination to one goal. Women were more openly talking about infertility with others, whereas most of the men were more restrained and preferred to talk to their partners. Sadness was the most dominant reaction among women regarding pregnancies in their close social environment, whereas helplessness was the most common reaction in men. Men were more ready to accept childlessness and more often believed in the success of treatment than women. In the assessment of infertility diagnosis and therapy, gender differences in the approach to disease should be taken into account. Psychological support should also be provided to the couples.

### Limitations

There are several possible limitations to this study, such as having a small study sample and low adherence to the study, being a single-center study, and usage of a nonvalidated questionnaire, which could make it impossible to generalize the findings to a whole population; further, the fact that the research concerns only couples who have taken up the treatment may also constitute a limitation.

## Figures and Tables

**Table 1:** Characteristics of the studied population.

Question	Women		Men	
	*n*	%	*n*	%
**Age**				
20–30 years	20	32.8	19	31.2
31–40 years	40	65.6	36	59.0
>40 years	1	1.6	6	9.8
**Education**				
Primary	-	-	1	1.6
Vocational	4	6.5	8	13.1
Secondary	13	21.3	20	32.8
Higher	44	72.2	32	52.5
**Place of residence**				
Urban	41	67.2	41	67.2
Rural	20	32.8	20	32.8
**Attitude to religion**				
A believer of Roman Catholic denomination	61 -	100 -	61 -	100 -
**Children**				
Yes	2	3.2	-	-
No	59	96.8	61	100

**Table 2:** Reproductive history of the surveyed couples.

Question	*n*	%
**Duration of the relationship**		
2–5 years	27	44.3
6–10 years	28	45.9
>10 years	6	9.8
**Duration of attempts to conceive**		
1–5 years	49	80.3
6–10 years	12	19.7
**The cause of infertility**		
Diagnosed	38	62.3
Not diagnosed	23	37.7
**ART methods used so far**		
IUI	40	65.6
IVF	3	4.9
IVF ICSI	5	8.2
None	13	21.3
**Previously used methods of contraception**		
Natural methods	6	9.8
Mechanical methods	7	11.5
Pharmacological methods	15	24.6
None	33	54.1
**Parity**		
Yes	2	3.2
No	59	96.8
**Miscarriage**		
Yes	7	11.5
No	54	88.5
**Is the cost of the treatment a problem?**		
Yes	47	77.1
No	14	22.9

ART—Assisted Reproduction Techniques, IUI—Intrauterine Insemination, IVF—In vitro Fertilization, ICSI—Intracytoplasmic Sperm Injection.

**Table 3:** The approach of men and women to the disease.

Variable	Women		Men		*p*
	*n*	%	*n*	%	
**Problems with procreation in the family**					0.050 *
Yes	5	8.2	4	6.6	
No	54	88.5	47	77.1	
Don’t know	2	3.3	10	16.4	
**Reaction to the diagnosis**					0.282
Sadness	20	32.8	21	34.4	
Grief	12	19.7	7	11.5	
Anxiety and fear	14	22.9	9	14.8	
Helplessness	10	16.4	11	18.0	
Anger	3	4.8	6	9.8	
Disappointment	2	3.3	7	11.5	
**Influence of diagnosis on relationship**					0.328
Grows apart	1	1.6	3	4.8	
Brings closer	15	24.6	18	29.5	
Nothing changed	25	49.0	28	45.9	
Hard to say	20	32.8	12	19.7	
**Difficulties during treatment**					0.021 *
Economic barrier	9	14.8	5	8.2	
Long procedures	10	16.4	21	34.4	
Subordination to one goal	26	42.6	12	19.7	
Pressure of the environment/relatives	5	8.2	10	16.4	
Embarrassing tests	11	18.0	13	21.3	
**Who do you talk to openly?**					<0.001 *
Spouse/partner	26	42.6	42	68.8	
Parents	-	-	6	9.8	
Siblings	11	18.0	2	3.2	
Friends	16	26.2	1	1.6	
Doctor	8	13.1	10	16.4	
**Does the family accept treatment?**					0.396
Yes	36	59.0	30	49.2	
No	1	1.6	3	4.9	
I don’t know	24	39.4	28	45.9	
**Does the family accept ART methods?**					<0.001 *
Yes	30	49.1	21	34.4	
No	14	23.0	37	60.7	
I don’t know	17	27.9	3	4.9	
**Do you accept all ART methods?**					0.979
Yes, all of them	43	70.5	44	72.1	
Not all of them	1	1.6	3	4.9	
None	17	27.9	16	26.3	
**Which ART method you do not accept?**					0.289
IUI	-	-	3	4.9	
IVF	8	13.1	5	8.2	
ICSI	10	16.4	9	14.8	
I accept all of them	43	70.5	44	72.1	
**Did the diagnosis affect your lifestyle?**					0.274
Yes	34	55.7	27	44.3	
No	27	44.3	34	55.7	
**Reaction to pregnancy/childbirth among relatives**					<0.001 *
Sadness	25	41.0	13	21.3	
Joy	12	19.6	11	18.0	
Jealousy	9	14.8	2	3.2	
Anger	5	8.2	1	1.6	
Helplessness	-	-	11	18.0	
Indifference	-	-	5	8.2	
Other	10	16.4	18	29.5	
**Readiness for childlessness**					0.050 *
Yes	15	24.6	25	41.0	
No	20	32.8	10	16.4	
I don’t know	26	42.6	26	42.6	
**Would you decide to adopt?**					0.474
Yes	22	36.1	16	26.2	
No	9	14.8	9	14.8	
I don’t know	30	49.1	36	59.0	
**Is psychological assistance needed?**					0.166
Yes, for all	26	42.6	21	34.4	
Only in justified cases	35	57.4	37	60.7	
No	-	-	3	4.9	
**What is your attitude to treatment?**					0.017 *
I believe in success	46	75.4	51	83.6	
I don’t believe, but I want to try	15	24.6	6	9.8	
I do it only for my partner	-	-	4	6.6	

* statistically significant.
